# Supplementary material for: Inhalation of Ultrafine Carbon Black-Induced Mitochondrial Dysfunction in Mouse Heart Through Changes in Acetylation
Source: Cells. 2025 Nov 4;14(21):1728. doi: 10.3390/cells14211728 (PMC12607619; doi:10.3390/cells14211728)
Supplement: Supplementary file 1 [file cells-14-01728-s001.zip › cells-3891709-supplementary.pdf]

Supplemental 1

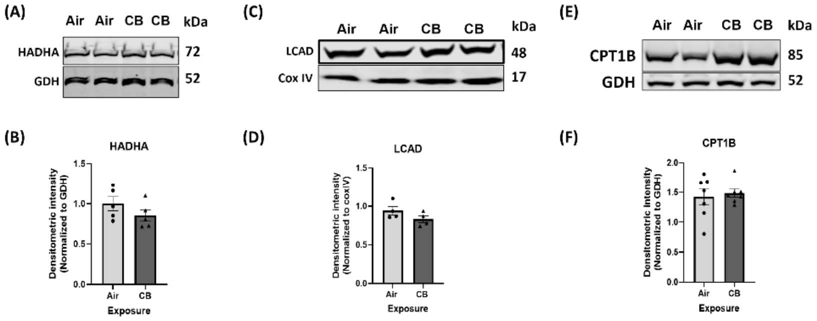

Supplemental Figure 1. Repeated CB exposure does not alter FAO proteins content.

(A–F) Western blot analyses and densitometry of key FAO enzymes: HADHA (n=5), LCAD; n=4, and CPT1b; n=7. Values are normalized to GDH or COXIV and expressed as mean ± SEM.

Supplemental 2

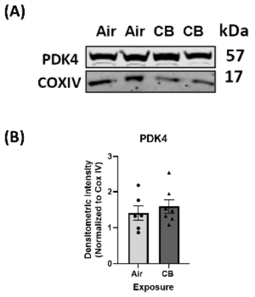

Supplemental Figure 2. Repeated CB exposure does not alter PDK4 protein content.

(A–D) Western blot analyses and densitometry of PDK4 enzyme; n=6. Values are normalized to COXIV and expressed as mean ± SEM.

Supplemental 3

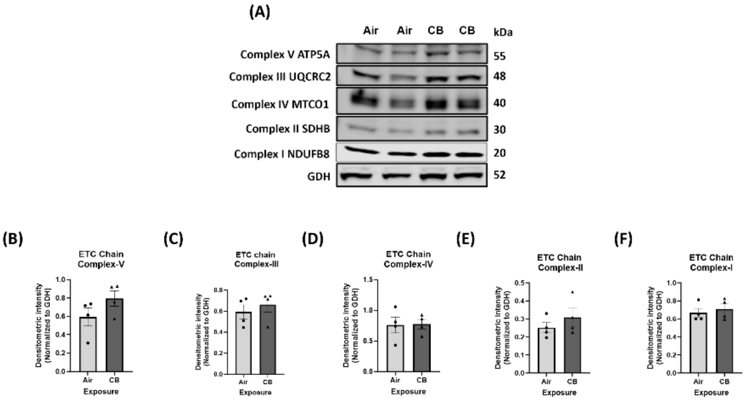

Supplemental Figure 3. Mitochondrial ETC protein expressions are unchanged under repeated CB exposure.

(A–F) Western blot and densitometric analysis of ETC Complex I–V subunits (complex I protein-NDUFB8, complex II protein-SDHB, complex III protein-UQCRC2, complex IV protein-MTCO1, complex V protein-ATP5A); n=4. Values are normalized to GDH and expressed as mean ± SEM.

Supplemental 4

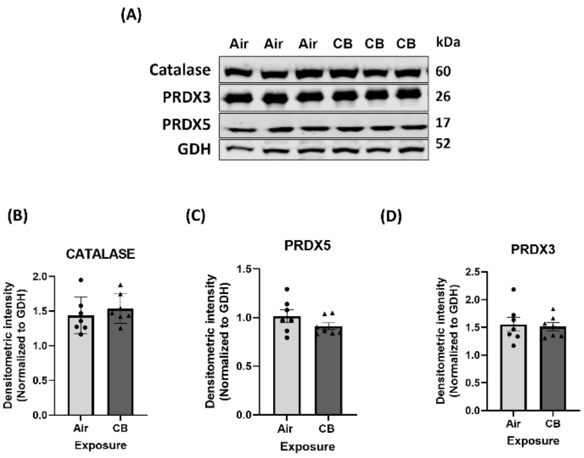

Supplemental Figure 4.

Antioxidant protein expressions are unchanged under repeated CB exposure.

(A–D) Western blot and densitometric analyses of mitochondrial antioxidants catalase, PRDX3, and PRDX5 protein expression; n=7.
